# Circulating N-terminal brain natriuretic peptide and cardiac function in response to acute systemic hypoxia in healthy humans

**DOI:** 10.1186/1479-5876-12-189

**Published:** 2014-07-03

**Authors:** Ilkka Heinonen, Matti Luotolahti, Olli Vuolteenaho, Mikko Nikinmaa, Antti Saraste, Jaakko Hartiala, Juha Koskenvuo, Juhani Knuuti, Olli Arjamaa

**Affiliations:** 1 Turku PET Centre, University of Turku and Turku University Hospital, PO Box 52, FI-20521 Turku, Finland; 2 Research Center of Applied and Preventive Cardiovascular Medicine, University of Turku and Turku University Hospital, PO Box 52, FI-20521 Turku, Finland; 3 Heart Center, University of Turku and Turku University Hospital, PO Box 52, FI-20521 Turku, Finland; 4 Department of Clinical Physiology and Nuclear Medicine, University of Turku and Turku University Hospital, PO Box 52, FI-20521 Turku, Finland; 5 Department of Biology, University of Turku and Turku University Hospital, PO Box 52, FI-20521 Turku, Finland; 6 Institute of Biomedicine, University of Oulu, Oulu, Finland; 7 Division of Experimental Cardiology, Thoraxcenter, Erasmus MC, University Medical Center Rotterdam, Rotterdam, The Netherlands

**Keywords:** Hypoxia, NT-proBNP, Cardiac function, Humans

## Abstract

**Background:**

As it remains unclear whether hypoxia of cardiomyocytes could trigger the release of brain natriuretic peptide (BNP) in humans, we investigated whether breathing normobaric hypoxic gas mixture increases the circulating NT-proBNP in healthy male subjects.

**Methods:**

Ten healthy young men (age 29 ± 5 yrs, BMI 24.7 ± 2.8 kg/m^2^) breathed normobaric hypoxic gas mixture (11% O_2_/89% N_2_) for one hour. Venous blood samples were obtained immediately before, during, and 2 and 24 hours after hypoxic exposure. Cardiac function and flow velocity profile in the middle left anterior descending coronary artery (LAD) were measured by Doppler echocardiography.

**Results:**

Arterial oxygen saturation decreased steadily from baseline value of 99 ± 1% after the initiation hypoxia challenge and reached steady-state level of 73 ± 6% within 20–30 minutes. Cardiac output increased from 6.0 ± 1.2 to 8.1 ± 1.6 L/min and ejection fraction from 67 ± 4% to 75 ± 6% (both p < 0.001). Peak diastolic flow velocity in the LAD increased from 0.16 ± 0.04 to 0.28 ± 0.07 m/s, while its diameter remained unchanged. In the whole study group, NT-proBNP was similar to baseline (60 ± 32 pmol/ml) at all time points. However, at 24 h, concentration of NT-proBNP was higher (34 ± 18%) in five subjects and lower (17 ± 17%), p = 0.002 between the groups) in five subjects than at baseline.

**Conclusion:**

In conclusion, there is no consistent increase in circulating NT-proBNP in response to breathing severely hypoxic normobaric gas mixture in healthy humans, a possible reason being that the oxygen flux to cardiac myocytes does not decrease because of increased coronary blood flow. However, the divergent individual responses as well as responses in different cardiac diseases warrant further investigations.

## Introduction

Natriuretic peptides are endogenous peptide hormones derived from the myocardium. They have autoregulatory effects to the heart by preventing atrial stretch via regulation of sodium and water balance but they also regulate directly blood vessel tonus and whole body energy homeostasis [1]. Especially brain natriuretic peptide (BNP), which possesses significant cardioprotective properties, is also frequently measured in patients with cardiovascular diseases as it provides diagnostic and prognostic value in many clinical scenarios. BNP is generally regarded to be released from the ventricles of the heart in response to volume or pressure overload. Emerging evidence from cell culture studies and animal experiments, however, suggests that also hypoxia [2] of cardiomyocytes could trigger the release of BNP [3,4]. It has also been shown with cultured human myocardial cells that hypoxia can induce BNP release [5]. Hypoxia effect can be separated from cardiomyocyte stretch [6], although not all human cell culture studies have repeated this response [7].

The physiological *in vivo* relevance of these cell culture findings remains unclear, however, as tissue oxygen levels may never reach such low and long-standing severe hypoxia values as is generally used in vitro studies. Nevertheless, normobaric hypoxic exposure for 60 minutes has been shown to induce slight increase in N-terminal BNP, but this study was performed in highly selected group of subjects that showed either high or low renin-angiotensin system activity [8]. On the other hand, a very severe (corresponding to altitude of 9144 m), but short (1–3 min) exposure to hypobaric hypoxia did not cause an increase in BNP levels [9]. Similarly, also a short hypobaric chamber exposure (25 min) at rest followed by 1 min of exercise did not lead to immediate increase in circulating BNP in healthy humans [10].

However, it might be that as BNP peptide is stored only in small amounts in secretary granules of ventricular myocytes [11], increase in BNP becomes apparent only after sufficient time period following hypoxia exposure, when gene expression has been triggered by hypoxia to form more of the peptide. Furthermore, cardiac function and coronary blood flow responses have not been monitored simultaneously with hypoxic exposure in these previous human studies, making it difficult to evaluate whether loading conditions for instance are affected and contribute to hypoxia-induced BNP release. Consequently, to elucidate these yet remaining aspects of the possible hypoxia-induced BNP release in humans in vivo, we measured circulating N-terminal proBNP levels repeatedly during breathing severely hypoxic gas mixture, and 2 hours and 24 hours after the exposure. Furthermore, cardiac function was also assessed comprehensively and simultaneously during hypoxic exposure, to further understand the possible independent role of hypoxia to trigger BNP release from cardiomyocytes.

## Methods

### Subjects

Ten healthy Caucasian young men (age 29 ± 5 yrs, height 181 ± 4 cm, weight 81 ± 11 kg, BMI 24.7 ± 2.8 kg/m^2^) volunteered to participate in this study. The purpose, nature, and potential risks of the study were explained to the subjects before they gave their written informed consent to participate. The subjects were normotensive non-smokers with no history of hypercholesterolemia, not taking any medications and had never experienced angina symptoms. The normal health status of the subjects was confirmed by a medical doctor by clinical examination, ECG and cardiac echocardiography before the experiments. The study was performed according to the Declaration of Helsinki and was approved by the Ethics Committee of the Hospital District of South-West Finland.

### Hypoxic exposure and blood sampling

The subjects were requested to avoid strenuous physical exercise in the 48 h prior to the experiments. The hypoxic exposure was performed in a fasting state and always started around 9 AM with study preparations. In these preparations 12-lead ECG was positioned and an antecubital vein was cannulated for blood sampling. Venous blood samples were obtained before, and 2 and 24 hours after hypoxic exposure and analyzed by standard hospital practices. After initial preparations, subjects breathed normobaric hypoxic gas mixture (11% O_2_/89% N_2_) for one hour. Arterial oxygen saturation was continuously measured by pulse oximetry and valid plethysmogram signal was confirmed throughout the studies. All of these measurements were performed when subjects were resting supine.

### Analysis of BNP

Plasma samples (0.9 ml) were first concentrated by extraction with SepPak C18 cartidges, to enable the quantification of the very low levels of circulating BNP peptides in healthy young subjects. NT-proBNP concentrations were determined by immunoassay specific to human NT-proBNP10–29 as previously reported [12,13]. NT-proBNP is a product of the same precursor and therefore reflects the secretion of the biologically active peptide BNP.

### Echocardiography

Transthoracic 2 dimensional (2D) and Doppler echocardiographic studies were performed with Acuson Sequoia 512 (Siemens Medical Solutions, USA) equipped with 3V2c transducer at rest and 20 min after beginning of the hypoxia. The digitized images were stored in Syngo Dynamics-system (Siemens, Siemens Medical Solutions, USA) for later analysis. The study subjects were examined in a left lateral decubitus position. Left and right ventricular dimensions and left ventricular wall thickness and ejection fraction and left atrial dimension were measured in parasternal long axis view from 2-dimensionally guided M-mode tracings using second harmonic 4.25 MHz scanning frequency. Pulmonary artery flow velocity (pulsed wave Doppler) was measured in the parasternal short axis view at the level of aortic root. The mitral inflow velocities and blood flow velocities in pulmonary veins (pulsed wave Doppler), pressure gradient between right ventricle and right atria (continuous wave Doppler), tissue Doppler velocities of the mitral annulus and M-mode traced valvular ring amplitudes were all recorded from apical 4-Chamber view. Vena cava inferior was examined from the subcostal view. The mean of at least three consecutive cardiac cycles was always calculated and averaged.

Diameter and blood flow velocity (pulsed wave Doppler) were measured in the mid left anterior descending coronary artery using a modified parasternal short axis view focusing on the interventricular sulcus using 15 L8 linear transducer as described earlier [14,15].

### Statistical analysis

Statistical analysis was performed using SAS/STAT statistical software (version 9.2, SAS institute Inc., Cary, NC, USA). Two-tailed Student’s t-test or one-way (or two-way in terms of group differences) ANOVA for repeated measurements was used for the analysis of statistical differences when appropriate. ANOVA was followed by Tukey post-hoc test if necessary to detect differences in different study time points, or groups. P values < 0.05 were considered statistically significant. All data are shown as mean ± SD.

## Results

**Figure 1:**
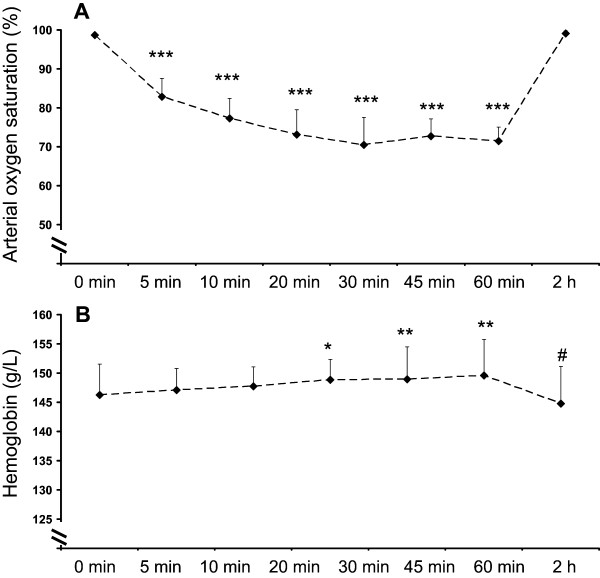
**Arterial oxygen saturation, as determined by pulse oxymetry (A), and venous haemoglobin concentration (B) before hypoxia (0 min), repeatedly during 60 min of hypoxic exposure, and 2 hours after the exposure.** *p < 0.05, **p < 0.01, and ***p < 0.001 compared to 0 min and 2 h, #p < 0.05 compared to 60 min.

**Figure 2:**
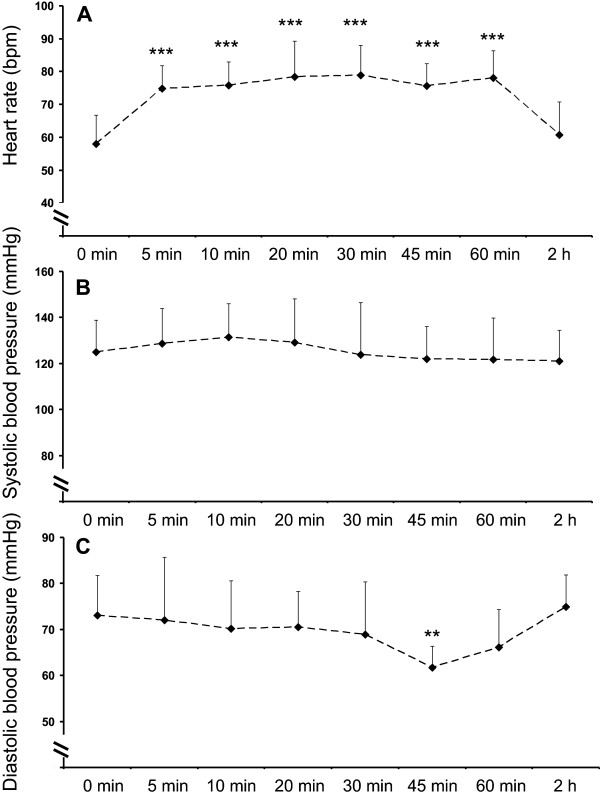
**Heart rate (A), and systolic (B) and diastolic blood pressures (C) before hypoxia (0 min), repeatedly during 60 min of hypoxic exposure, and 2 hours after the exposure.** **p < 0.01, and ***p < 0.001 compared to 0 min and 2 h.

All of the subjects had normal baseline arterial oxygen saturation of 99 ± 1% at baseline, which decreased steadily after the initiation of hypoxic breathing and reached a stable steady-state level (73 ± 6%) within 20–30 min after the initiation of hypoxic exposure based on continuous pulse oximetry (Figure 1A). This level of oxygen saturation was maintained until the removal of the hypoxic mask after 1 hour, after which saturation reached normal, pre-hypoxic values (99 ± 1%) within 1 min (Figure 1A). Hemoglobin concentration was increased towards the end of the hypoxic exposure, but returned to pre-hypoxia levels after 2 hours of returning to normoxic conditions (Figure 1B).Heart rate response followed a similar, but reversed pattern to that of arterial oxygen saturation, being 58 ± 9 bpm at resting baseline, 79 ± 11 during hypoxia, and 61 ± 1 bpm two hours after the end of hypoxic exposure (Figure 2A). Systolic blood pressure did not change (Figure 2B) in response to hypoxia (p = 0.42 in ANOVA), but diastolic blood pressure was slightly lower in the end of hypoxia than at baseline or during normoxic recovery from the hypoxia exposure (Figure 2C).

**Figure 3:**
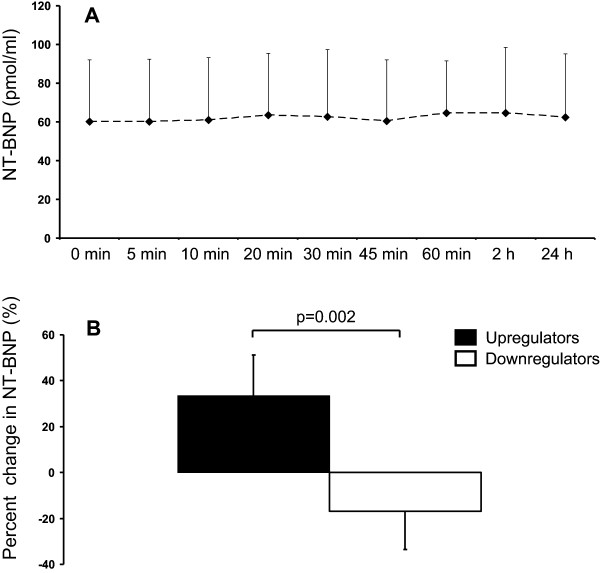
**NT-proBNP responses.** Circulating NT-proBNP concentration **(A)** before hypoxia (0 min), repeatedly during 60 min of hypoxic exposure, and 2 and 24 hours after the exposure. In panel **B** mean per cent change in NT-proBNP of two divergent groups (n=5 in both groups) are presented, which showed either increase (“upregulators”) or reduction (“downregulators”) from baseline to 24 hours of hypoxic exposure in NT-proBNP concentration.

In Table 1 parameters describing cardiac function at baseline and in hypoxia are presented. No regional wall motion abnormalities were detected. Of note, hypoxia led to increased cardiac output and ejection fraction, but atrial sizes did not change. Left coronary artery blood velocity was also increased, but its diameter remained unchanged in response to hypoxia. A decrease in E/A-ratio was also observed.At the whole study group level there was no change in NT-proBNP concentration in response to applied systemic hypoxia (Figure 3A). However, two distinct NT-proBNP response groups to hypoxia were also identified, as in five subjects the 24 h concentration of NT-proBNP was elevated on average 34 ± 18% as compared with the baseline concentration, while in five subjects it was reduced by an average of 17 ± 17% lower (Figure 3B). NT-proBNP either increased or decreased in all of these five subjects. Mean heart rate was consistently higher throughout the experiment in the five subjects with elevated BNP at 24 hours (62 ± 11, 77 ± 8, 78 ± 9, 81 ± 10, 84 ± 7, 78 ± 8, 81 ± 6, 65 ± 11 bpm at baseline before hypoxia, and 5, 10, 20, 30, 45, 60 min time points during, and 2 hours after hypoxia, respectively) as compared to the other five individuals (55 ± 5, 73 ± 6, 74 ± 5, 76 ± 12, 73 ± 9, 73 ± 5, 76 ± 11, 56 ± 6, respectively, p < 0.01 for the group difference in two-way ANOVA, hypoxia*group interaction p = NS), but there were no group differences, or even trends towards differences, in arterial oxygen saturation, haemoglobin concentration, blood pressures or any of the echocardiography variables measured in the present study (data not shown).

**Table 1:** Cardiac function at baseline and after 20 minutes exposure to hypoxic stimulus

	**Baseline**	**Hypoxia**
Cardiac output (L/min)	6.0 ± 1.2	8.1 ± 1.6***
Ejection fraction (%)	67 ± 4	75 ± 6***
RV ED diameter (mm)	25 ± 4	26 ± 3
RA diameter (mm)	20 ± 4	19 ± 2
LA diameter (mm)	39 ± 4	38 ± 3
PA blood flow velocity (m/s)	0.8 ± 0.1	1.0 ± 0.1*
Vena cava inferior (mm)	16.8 ± 4.6	15.2 ± 4.2
RV-to-RA pressure gradient (mmHg)	28 ± 5	31 ± 3*
RV E’/ A’ (cm/s)	1.5 ± 0.4	1.4 ± 0.2
RV S_m_ (cm/s)	0.20 ± 0.04	0.24 ± 0.04*
Pulmonary vein s/d ratio	1.3 ± 0.2	1.4 ± 0.3
TAPSE (cm)	2.6 ± 0.2	3.0 ± 0.3**
LAD diameter (cm)	5.62 ± 0.64	5.60 ± 0.64
LAD blood flow velocity (m/s)	0.16 ± 0.4	0.28 ± 0.07**
LV lateral wall E’/A’	1.9 ± 0.7	1.5 ± 0.6
LV septal wall E’/A’	1.6 ± 0.3	1.5 ± 0.3
LV septum S_m_ (cm/s)	0.12 ± 0.02	0.15 ± 0.05
LV lateral wall S_m_ (cm/s)	0.16 ± 0.06	0.19 ± 0.06
Lateral mitral annular displacement (cm)	1.5 ± 0.2	1.8 ± 0.2**
Septal mitral annular displacement (cm)	1.5 ± 0.1	1.7 ± 0.3*
E/A –ratio	1.6 ± 0.3	1.4 ± 0.2*

LV = left ventricle, RV = Right ventricle, LA = left atrium , RA = right atrium, TAPSE = Tricuspid annular plane systolic excursion, PA = pulmonary artery, E’ = tissue-Doppler early diastolic velocity, A’ = tissue-Doppler late diastolic velocity, S_m_ = tissue-Doppler systolic velocity, E = Mitral inflow early peak velocity, A = Mitral inflow atrial peak velocity. *p < 0.05, **p < 0.01, ***p < 0.001 compared to baseline. LAD = the left anterior descending coronary artery.

## Discussion

In the present study we measured cardiac function, coronary blood flow velocity and peripheral circulating levels of NT-proBNP in response to breathing severely hypoxic gas mixture in healthy young male volunteers to study the possibility that hypoxia per se could trigger the release of NT-proBNP. We did not find any consistent increase in circulating NT-proBNP, which was likely due to the fact that despite the marked reduction of oxygen saturation, oxygen supply to the cardiomyocytes was fully compensated for by the increased coronary blood flow in these healthy young adults. Nevertheless, two distinct NT-proBNP response groups to hypoxia were also identified in the present study. It will be discussed that this divergent response is likely related to hypoxic regulation of BNP genetic responses, and in the long term might determine individual adaptation to chronic hypoxia.

Our findings in terms of cardiac responses to acute systemic hypoxia are well in accordance with the established literature, which also describes tachycardia and largely unchanged blood pressure as one of the key feature of hypoxic physiological response [16-18]. Although hypoxia suppresses myocardial contractility in some studies that have been performed in vitro, also our finding of higher ejection fraction, thus improved emptying of the ventricle, supports the notions that hypoxia may induce compensatory improvement in cardiac pump function in vivo in healthy humans [16,19]. Moreover, although a decrease in early-to-late filling (E/A-ratio) of the ventricle was also observed in the present study, which would normally suggest impaired diastolic filling, it has been previously documented that this is largely the result of decreased early filling due to preload reduction, while there is simultaneously a greater contribution of atrial contraction in acute systemic hypoxia [16].

However, when it comes to the NT-proBNP levels during acute hypoxic exposure, as sampled repeatedly during the exposure, as well as the 2 and 24 h level after hypoxic exposure, we observed that it remained essentially similar to the pre-exposure baseline NT-proBNP level. This was the case even when the arterial oxygen saturation dropped close to 70% during the severe acute hypoxic exposure. However, despite this reduction it is likely that absolute oxygen supply to heart was not actually decreased, as coronary blood flow velocity increased by 75% while the diameter of left anterior coronary artery remained unchanged. Therefore, it is apparent that the investigation of BNP release is hampered by the fact that despite the reduction of oxygen saturation, oxygen supply of the cardiomyocytes is mostly fully compensated by the increased coronary blood flow in healthy young adults.

From the present results it is clear that breathing severely hypoxic gas mixture does not elicit increased NT-proBNP level, as despite the decreased arterial oxygen saturation cardiac myocytes can obtain unchanged (or even increased) amount of oxygen as myocardial blood flow increases markedly [20]. Myocardial hypoxia may however occur if coronary blood flow cannot respond to reduced oxygen saturation or oxygen demand is excessively increased, as might happen in many cardiac patients in which BNP is frequently determined as a diagnostic measure. Local cardiac hypoxia typical for many cardiac patients may cause the increase in circulating NT-proBNP levels, in addition to well-established causes of pressure and volume overload [21-23], but unfortunately, ischemia-prone individuals were not investigated in the present study and this aspect warrants further experimentation. Another possible explanation in addition to the blood flow compensation is that our one-hour hypoxic exposure was too short to induce the expression of BNP gene. In long-term experiments circulating BNP has been shown to be increased especially in subjects who suffer from chronic mountain sickness [24-26], but clear interpretation if hypoxia per se triggers BNP release remains open, as these studies are also confounded by several other stress factors, such as physical stress caused by hiking to and on the mountains and not be hypoxia per se. These findings have neither been confirmed by all studies [27]. Preoperative BNP levels are also within normal levels in patients with tetralogy of Fallot (severe congenital cyanotic heart defect) who have generally at least moderate decreases in systemic oxygen saturation [28].

Finally, despite the fact that no increase in mean levels of NT-proBNP was detected in the present study, we observed two differently responding groups; in five subjects, the 24 h concentration of NT-proBNP was higher than the baseline concentration, while in five subjects it was lower. As these divergent responses could not be related to changes in cardiac function during hypoxia, it is possible that these differing responses are due to differences in single nucleotide polymorphisms in gene areas regulating BNP expression. In this regard it is interesting that BNP gene is under the direct control of HIF (hypoxia-inducible factor) [5,7,29], which is the master regulator of hypoxic responses. Thus, it remains to be investigated whether these two divergent BNP responses could be localized to differences in single nucleotide polymorphisms that are under the control of HIF.

Moreover, it might also be that these differences in NT-BNP responses also determine the susceptibility to hypoxic mountain sickness [24-26]. In this regard, circulating BNP has been shown by Ge and Mo et al. to be predictive of chronic mountain sickness [26]. However, in their study patients with mountain sickness also had higher pulmonary artery pressures and lower arterial oxygen saturations compared to subjects who did not get altitude sickness [26]. Therefore, it might be that also in this case the increase in BNP is just a result of increased cardiac pressures [21-23], but further studies are still warranted to investigate the emerging evidence from animal studies that hypoxia, and/or ischemia, per se could trigger the release of brain natriuretic peptides also in humans.

### Methodological considerations

It is a limitation that we did not measure NT-proBNP levels directly from coronary sinus, but instead peripheral venous site was used for sampling. Together with its binding to receptors, this may have caused NT-proBNP to be diluted to body fluids and lowered the detection limit to observe changes. However, not even a trend towards changes was observed as whole study group level, although on the other hand changes were robust when investigated at the sub-group level indicating the validity of peripheral sampling. Moreover, circulating levels of BNP show a close relationship with its content in myocardium [30], making its measurement from peripheral blood valid approach, especially in healthy volunteers who have no medical or other reasons for cardiac catheterization.

In conclusion, we did not find increase in circulating NT-BNP in response to breathing severely hypoxic gas mixture in humans in the present study. However, cardiac myocytes did not probably experience reduced amount of oxygen, as despite the fact that arterial oxygen saturation was markedly reduced, oxygen supply to the cardiomyocytes was likely fully compensated for by the increased coronary blood flow in the studied healthy young adults. Acute studies patients with cardiac diseases, especially those with limited possibility to adjust coronary blood flow, and chronic hypoxic exposure studies in healthy humans under well-controlled conditions are required to conclusively show the role of hypoxia in triggering NT-BNP release.

## Competing interests

The author declares that they have no competing interests.

## Authors’ contributions

All authors have participated in study planning, performing and writing the manuscript. All have read and approved submission of the final version of the manuscript.

## References

[B1] Moro C Lafontan M Natriuretic peptides and cGMP signaling control of energy homeostasis Am J Physiol Heart Circ Physiol 2013 304 H358 H368 10.1152/ajpheart.00704.2012 23203965

[B2] Nikinmaa M “What is hypoxia?” Acta Physiol (Oxf) 2013 doi:10.1111/apha.12146 10.1111/apha.12146 23834481

[B3] Arjamaa O Nikinmaa M Hypoxia regulates the natriuretic peptide system Int J Physiol Pathophysiol Pharmacol 2011 3 191 201 21941610 PMC3175745

[B4] Arjamaa O Nikinmaa M Natriuretic peptides in hormonal regulation of hypoxia responses Am J Physiol Regul Integr Comp Physiol 2009 296 R257 R264 19005014 10.1152/ajpregu.90696.2008

[B5] Casals G Ros J Sionis A Davidson MM Morales-Ruiz M Jimenez W Hypoxia induces B-type natriuretic peptide release in cell lines derived from human cardiomyocytes Am J Physiol Heart Circ Physiol 2009 297 H550 H555 10.1152/ajpheart.00250.2009 19542490

[B6] Mollmann H Nef HM Kostin S Dragu A Maack C Weber M Troidl C Rolf A Elsasser A Bohm M Brantner R Hamm CW Holubarsch CJ Ischemia triggers BNP expression in the human myocardium independent from mechanical stress Int J Cardiol 2010 143 289 297 10.1016/j.ijcard.2009.03.012 19329198

[B7] Luo Y Jiang C Belanger AJ Akita GY Wadsworth SC Gregory RJ Vincent KA A constitutively active hypoxia-inducible factor-1alpha/VP16 hybrid factor activates expression of the human B-type natriuretic peptide gene Mol Pharmacol 2006 69 1953 1962 10.1124/mol.105.017905 16507742

[B8] Due-Andersen R Pedersen-Bjergaard U Hoi-Hansen T Olsen NV Kistorp C Faber J Boomsma F Thorsteinsson B NT-pro-BNP during hypoglycemia and hypoxemia in normal subjects: impact of renin-angiotensin system activity J Appl Physiol 2008 104 1080 1085 10.1152/japplphysiol.01082.2007 18258801

[B9] Karadag R Sen A Yildirim N Basmak H Golemez H Cakir E Akin A The relation between intraocular pressure change and plasma natriuretic peptide under simulated hypobaric conditions Indian J Ophthalmol 2010 58 195 198 10.4103/0301-4738.62642 20413920 PMC2886248

[B10] Woods D Hooper T Mellor A Hodkinson P Wakeford R Peaston B Ball S Green N Brain natriuretic peptide and acute hypobaric hypoxia in humans J Physiol Sci 2011 61 217 220 10.1007/s12576-011-0141-3 21431981 PMC10717752

[B11] Levin ER Gardner DG Samson WK Natriuretic peptides N Engl J Med 1998 339 321 328 10.1056/NEJM199807303390507 9682046

[B12] Ala-Kopsala M Magga J Peuhkurinen K Leipala J Ruskoaho H Leppaluoto J Vuolteenaho O Molecular heterogeneity has a major impact on the measurement of circulating N-terminal fragments of A- and B-type natriuretic peptides Clin Chem 2004 50 1576 1588 10.1373/clinchem.2004.032490 15265819

[B13] Ala-Kopsala M Moilanen AM Rysa J Ruskoaho H Vuolteenaho O Characterization of molecular forms of N-terminal B-type natriuretic peptide in vitro Clin Chem 2010 56 1822 1829 10.1373/clinchem.2010.148775 20926601

[B14] Kiviniemi TO Saraste M Koskenvuo JW Airaksinen KE Toikka JO Saraste A Parkka JP Hartiala JJ Coronary artery diameter can be assessed reliably with transthoracic echocardiography Am J Physiol Heart Circ Physiol 2004 286 H1515 H1520 14656707 10.1152/ajpheart.00819.2003

[B15] Kiviniemi TO Toikka JO Koskenvuo JW Saraste A Saraste M Parkka JP Raitakari OT Hartiala JJ Vasodilation of epicardial coronary artery can be measured with transthoracic echocardiography Ultrasound Med Biol 2007 33 362 370 10.1016/j.ultrasmedbio.2006.08.012 17188799

[B16] Naeije R Physiological adaptation of the cardiovascular system to high altitude Prog Cardiovasc Dis 2010 52 456 466 10.1016/j.pcad.2010.03.004 20417339

[B17] Hultgren HN Grover RF Circulatory adaptation to high altitude Annu Rev Med 1968 19 119 152 10.1146/annurev.me.19.020168.001003 4871683

[B18] Bartsch P Gibbs JS Effect of altitude on the heart and the lungs Circulation 2007 116 2191 2202 10.1161/CIRCULATIONAHA.106.650796 17984389

[B19] Dedobbeleer C Hadefi A Naeije R Unger P Left ventricular adaptation to acute hypoxia: a speckle-tracking echocardiography study J Am Soc Echocardiogr 2013 26 7 736 745 10.1016/j.echo.2013.04.012 23706341

[B20] Namdar M Koepfli P Grathwohl R Siegrist PT Klainguti M Schepis T Delaloye R Wyss CA Fleischmann SP Gaemperli O Kaufmann PA Caffeine decreases exercise-induced myocardial flow reserve J Am Coll Cardiol 2006 47 405 410 10.1016/j.jacc.2005.08.064 16412869

[B21] Tenhunen O Szokodi I Ruskoaho H Posttranscriptional activation of BNP gene expression in response to increased left ventricular wall stress: role of calcineurin and PKC Regul Pept 2005 128 187 196 10.1016/j.regpep.2004.12.024 15837527

[B22] Maeder MT Mariani JA Kaye DM Hemodynamic determinants of myocardial B-type natriuretic peptide release: relative contributions of systolic and diastolic wall stress Hypertension 2010 56 682 689 20713912 10.1161/HYPERTENSIONAHA.110.156547

[B23] Wiese S Breyer T Dragu A Wakili R Burkard T Schmidt-Schweda S Fuchtbauer EM Dohrmann U Beyersdorf F Radicke D Holubarsch CJ Gene expression of brain natriuretic peptide in isolated atrial and ventricular human myocardium: influence of angiotensin II and diastolic fiber length Circulation 2000 102 3074 3079 10.1161/01.CIR.102.25.3074 11120697

[B24] Woods D Hooper T Hodkinson P Ball S Wakeford R Peaston B Bairsto C Green N Mellor A Effects of altitude exposure on brain natriuretic peptide in humans Eur J Appl Physiol 2011 111 2687 2693 10.1007/s00421-011-1881-8 21394641

[B25] Woods DR Begley J Stacey M Smith C Boos CJ Hooper T Hawkins A Hodkinson P Green N Mellor A Severe acute mountain sickness, brain natriuretic peptide and NT-proBNP in humans Acta Physiol (Oxf) 2012 205 349 355 10.1111/j.1748-1716.2012.02407.x 22222437

[B26] Ge RL Mo VY Januzzi JL Jin G Yang Y Han S Wood MJ Levine BD B-type natriuretic peptide, vascular endothelial growth factor, endothelin-1, and nitric oxide synthase in chronic mountain sickness Am J Physiol Heart Circ Physiol 2011 300 H1427 H1433 10.1152/ajpheart.00366.2010 21217075

[B27] Toshner MR Thompson AA Irving JB Baillie JK Morton JJ Peacock AJ NT-proBNP does not rise on acute ascent to high altitude High Alt Med Biol 2008 9 307 310 10.1089/ham.2008.1054 19115915

[B28] Koch A Zink S Singer H B-type natriuretic peptide in paediatric patients with congenital heart disease Eur Heart J 2006 27 861 866 10.1093/eurheartj/ehi773 16467354

[B29] Weidemann A Klanke B Wagner M Volk T Willam C Wiesener MS Eckardt KU Warnecke C Hypoxia, via stabilization of the hypoxia-inducible factor HIF-1alpha, is a direct and sufficient stimulus for brain-type natriuretic peptide induction Biochem J 2008 409 233 242 10.1042/BJ20070629 17822384

[B30] Goetze JP Christoffersen C Perko M Arendrup H Rehfeld JF Kastrup J Nielsen LB Increased cardiac BNP expression associated with myocardial ischemia FASEB J 2003 17 1105 1107 12709407 10.1096/fj.02-0796fje

